# Proteomic profiling of Tau interactome in brain‐derived extracellular vesicles uncover key molecules contributing to tau pathology spread in Alzheimer's disease

**DOI:** 10.1002/alz70855_107077

**Published:** 2025-12-25

**Authors:** Tsuneya Ikezu, Zhengrong Zhang, Yang You, Kaiwen Yu, Arun Reddy Ravula, Yunfei I Chen, Bridgette Melvin, Alejandro Longtree‐Preciado, Seiko Ikezu, Michael A. DeTure, Dennis W. Dickson, Junmin Peng

**Affiliations:** ^1^ Mayo Clinic Florida, Jacksonville, FL, USA; ^2^ St. Jude Children's Research Hospital, Memphis, TN, USA; ^3^ Mayo Clinic Florida, JACKSONVILLE, FL, USA; ^4^ Mayo Clinic, Jacksonville, FL, USA

## Abstract

**Background:**

Brain cells secrete extracellular vesicles (EVs) containing signaling and pathological proteins related to Alzheimer's disease (AD) and related disorders. Recent studies demonstrate brain‐derived EVs (BDEVs) contain misfolded tau and are transmissible of tau pathology in the brain. However, the underlying mechanism regarding EV‐mediated tau pathology is still largely uncharacterized.

**Method:**

We conducted the immuno‐affinity purification of tau in human brain‐derived EVs (BDEVs) isolated from age and sex‐matched 14 AD and 14 CTRL cases and performed tandem mass‐tag mass spectrometry to profile unbiased tau interactome in BDEVs. We used Nanotemper Monolith to validate the interaction of EV‐tau interactors with tau, and super‐resolution microscopy (Nanoimager) to detect their colocalization in a single‐EV level. We next designed siRNAs to silence EV‐tau interactors in SH‐SY5Y cells overexpressing human P301L tau (SH‐SY5Y‐P301Ltau) and assessed tau loading to EVs by Flow Nanoanalyzer. These EVs were tested for their uptake and tau seeding by human iPSC‐derived neurons (iNeurons). We finally tested the inhibitors of identified molecules on BDEV‐mediated tau propagation *in vivo*.

**Result:**

A total of 764 proteins were identified in BDEV‐associated tau interactome from CTRL and AD patients. Sixty‐five proteins were significantly downregulated in AD BDEVs; whereas 5 proteins were significantly upregulated in AD BDEVs compared to CTRL BDEVs. The most enriched tau‐interacting proteins were significantly positively correlated with Braak stage. We confirmed the direct binding of candidate proteins to tau with micromolar binding affinities by Monolith, and their colocalization with tau in human BDEVs using Nanoimager. Silencing of candidate molecules in human SH‐SY5Y‐P301Ltau cells showed reduced loading of tau in EVs, their reduced uptake by iNeurons, and tau seeding activities. Finally, intracerebroventricular injection of neutralizing antibody against the most promising target results in reduction of tau pathology in PS19 mice expressing P301S human tau mutant.

**Conclusion:**

Our study identified tau‐interacting molecules highly enriched in AD BDEVs compared to CTRL BDVs. Silencing of candidate molecule reduced tau loading to the EVs and their uptake by iNeurons and neutralizing antibody suppressed tau dissemination, highlighting them as promising therapeutic targets to halt tau pathology in AD and related tauopathy.

